# Impact of Mid-Luteal Phase GnRH Agonist Administration on Reproductive Outcomes in GnRH Agonist-Triggered Cycles: A Randomized Controlled Trial

**DOI:** 10.3389/fendo.2017.00124

**Published:** 2017-06-15

**Authors:** Abdelhamid Benmachiche, Sebti Benbouhedja, Abdelali Zoghmar, Amel Boularak, Peter Humaidan

**Affiliations:** ^1^Center for Reproductive Medicine, Clinique Ibn Rochd, Constantine, Algeria; ^2^The Fertility Clinic, Skive Regional Hospital, Skive, Denmark; ^3^Faculty of Health Aarhus University, Aarhus, Denmark

**Keywords:** GnRHa trigger, *in vitro* fertilization, luteal phase support, luteal GnRH agonist, mid-luteal steroids

## Abstract

**Objective:**

To explore whether the addition of a mid-luteal bolus of GnRH agonist (GnRHa) improves the implantation rate (IR) in *in vitro* fertilization (IVF) cycles.

**Design:**

A randomized controlled trial.

**Setting:**

Private IVF center.

**Patients:**

328 IVF/intracytoplasmic sperm injection patients were triggered with GnRHa and received 1,500 IU HCG on the day of oocyte pick-up (OPU) in addition to a standard luteal phase support (LPS).

**Intervention(s):**

In addition, the study group received a bolus of GnRHa 6 days after OPU, whereas the control group did not.

**Main outcome measure:**

Implantation rate.

**Secondary outcome measure(s):**

Ongoing pregnancy (OP) and live birth (LB) rates.

**Results:**

Although serum concentrations of FSH, LH, E2, and P on day OPU + 7 were significantly higher in the study group compared to the control group, the IR was not statistically different between the treatment group (27%) and the control group (23%) [odds ratio (OR) 1.2 (95% CI 0.9–1.7), *P* < 0.27]. Similarly, the OP rate was 37% in the treatment group and 31% in the control group [OR 1.3 (95% CI 0.8–2.0), *P* < 0.23]. The LB rate was 36% in the treatment group and 31% in the control group [OR: 1.3 (95% CI 0.8–2.0), *P* < 0.27].

**Conclusion:**

Although a trend toward a higher IR and pregnancy rate was observed in the treatment group, this difference was not statistically significant. However, the absolute risk difference of 5% found for LB is clinically relevant, warranting further investigation.

**NCT:**

02053779.

## Introduction

Initial studies in *in vitro* fertilization (IVF)/intracytoplasmic sperm injection (ICSI) patients have demonstrated that the use of a GnRH agonist (GnRHa) trigger, followed by fresh transfer and a standard luteal phase support (LPS) was associated with unacceptably high rates of loss in early pregnancy compared to hCG trigger, particularly in normal responder (NR) patients (1, 2). It has been subsequently concluded that this early pregnancy loss was caused by luteal phase (LP) insufficiency, despite the use of a standard LPS package of progesterone (P) and estradiol (E2). The LP defect was primarily caused by reduced early-mid-luteal luteinizing hormone (LH) activity, resulting in a significant reduction in progesterone output by the corpora lutea (CL) as no adverse effects were seen with respect to the maturity rate of oocytes, fertilization rates, embryo quality, and reproductive outcomes during the subsequent replacement of frozen embryos derived from women who had received a GnRHa trigger (3–7). Following these first disappointing results several studies have shown a LP rescue with the use of a modified LPS, resulting in reproductive outcomes comparable to those observed after hCG triggering (3, 4, 8–10). Up until now, the method of modified LPS reported by Humaidan et al. (3) appears to be the most frequently used regimen in fresh embryo transfer cycles (11). However, further fine-tuning of LPS after the GnRHa trigger could still possibly be warranted (12–14). Significantly increased implantation rates (IRs) were previously reported in oocyte recipients as well as in patients who were triggered with hCG, if they received a single mid-luteal bolus of GnRHa in addition to standard LPS (15–17). However, others have failed to support this finding (18, 19). Importantly, there are significant differences in the early-mid-LP endocrine pattern when GnRHa triggers and hCG triggers are compared, especially in terms of LH levels. From this, it could be hypothesized that the GnRHa-triggered IVF cycle could benefit more from the addition of a bolus of GnRHa to boost the circulating endogenous LH and thus, progesterone levels around the time of implantation than the hCG triggered cycle. No studies previously investigated this issue. Therefore, the aim here was to explore a possible fine-tuning of the LPS of GnRHa-triggered IVF/ICSI cycles, using the previously suggested protocol of Humaidan et al. (3). Here, we have combined a GnRHa trigger and the 1,500 IU HCG dose administered on the day of oocyte pick-up (OPU) (3, 4) with a single dose of GnRHa administered 6 days after OPU, i.e., 2 days before the expected day of implantation in an attempt to mimic the natural cycle peak of progesterone during the mid-LP. In addition, a standard LPS consisting of vaginal progesterone (P) and oral estrogen (E2) was used. By possibly optimizing the early and mid-LP in terms of steroids, it was also hypothesized that we would subsequently optimize IRs, which was the primary outcome measure of the study.

## Materials and Methods

### Study Design

This single-center, prospective, randomized, controlled trial was conducted from February 2014 to January 2016 at the IVF center Ibn Rochd, Constantine, Algeria. All patients provided written informed consent to participate in the study, which was conducted in accordance with the Declaration of Helsinki and Good Clinical Practice. The research project was approved by the Ethics Committee of the University Hospital Centre Ibn Badis, Constantine. Moreover, the study was registered in ClinTrial.gov, Number: 02053779.

### Study Participants

A total of 328 women were included in the study. Women were enrolled into the study if they fulfilled the following criteria: aged <40 years; baseline FSH < 12 IU/L; no uterine fibroids, Mullerian malformations, ovarian (endometrioma), or adnexal (hydrosalpinx) abnormalities. In addition, patients needed to have at least one embryo for fresh transfer. The exclusion criteria were a very high risk of ovarian hyper stimulation syndrome (OHSS) development (>30 follicles >11 mm on the day ovulation was triggered), poor responders identified according to Bologna criteria, or having a partner with azoospermia.

### Randomization

Women meeting the inclusion criteria were randomized per block of four patients according to a computer-generated randomization list. The randomization was performed by a nurse on the day of embryo transfer (ET) 2–3 days after OPU. The researchers were blinded to group allocations. Patients were assigned to one of two LPS groups. The study group received a single-dose of GnRHa (triptorelin 0.1 mg s.c.) on day 6 after OPU administered by either a nurse or by the patient herself, in addition to standard LPS; the control group received standard LPS, only.

### Hormonal Treatment and Ovulation Trigger

The ovarian stimulation was initiated from day 3 of the menstrual cycle and continued until the day that ovulation was induced. The standard daily starting dose of recombinant human FSH (Gonal-F^®^; Merck Serono) or (Puregon^®^; MSD) was 150–225 IU, depending on patient age, BMI, antral follicle count, and basal serum FSH levels. After 5 days, doses were adjusted according to ovarian response. Once the leading follicle had reached a size of 13 mm, co-treatment with a GnRH antagonist (Cetrotide^®^ 0.25 mg; Merck Serono) or (Orgalutran^®^ 0.25 mg; MSD) was initiated and continued up until and including the day of induction of ovulation. When at least three follicles had reached a size of 17 mm, ovulation induction was performed with a single bolus of 0.2 mg triptorelin, s.c. (Decapeptyl^®^ 0.1 mg, Ipsen, France), followed by OPU 36 h later. Retrieved oocytes were fertilized by either IVF or ICSI, depending on sperm quality.

### Embryo Transfer and LPS

According to the local regional policy and after an agreement between the patient and the medical team, one to three embryos were transferred.

For LPS, in addition to a bolus of hCG 1,500 IU, IM (Pregnyl^®^; MSD) given 1 h after OPU, all patients received micronized P (600 mg/day) vaginally (Utrogestan^®^; Laboratoires Besins-Iscovesco, Paris, France) and estradiol (4 mg/day) orally (Progynova^®^ 2 mg; Schering, Madrid, Spain), beginning on the day after oocyte retrieval and continuing until either a fetal heartbeat was detected on ultrasound at 5 weeks after OPU or a negative pregnancy test.

### Blood Samples and Hormone Assays

Blood sampling was performed for FSH, LH, TSH, E2, and prolactin on day 1 of stimulation, for E2, P, and LH on the day of ovulation induction, for E2, P, LH, and FSH on OPU day +7, within 14–16 h following the Triptorelin 0.1 mg injection particularly in the study group. Beta hCG was measured 14 days after OPU. Sera were analyzed immediately using a Vidas kit (BioMerieux, France). All measurements were performed according to the manufacturer’s instructions.

### Outcomes and Measures

The primary endpoint determining efficacy was IR. Secondary endpoints were the mid-luteal steroid levels, the rate of early pregnancy loss, the rate of clinical pregnancy, the rate of ongoing pregnancy (OP), the live birth (LB) rate, and the incidence of OHSS. The IR was defined as the number of gestational sacs with a fetal heartbeat, as assessed by ultrasound at 5 weeks after OPU, divided by the number of embryos transferred. Clinical pregnancy was defined as a positive serum β-hCG test with ultrasound evidence of a gestational sac and fetal heart beat at 5 weeks after OPU. OP was defined as pregnancy progressing beyond 12 weeks after OPU.

A LB was defined as a delivery of a live baby beyond 26 weeks after OPU. Early pregnancy loss was defined as a positive serum β-hCG test without ultrasound evidence of a gestational sac or a gestational sac without a fetal heartbeat.

### Sample Size and Power Calculation

Based on previously published data (15), which shows that the administration of a bolus of GnRHa 6 days after ICSI in oocyte recipients increases the IR from 25.1 to 36.9%, it was hypothesized that a similar increase might be expected in patients undergoing ovarian stimulation. Using these findings, the sample size was calculated using a two-sided significance level of 0.05 and a power of 80% to detect a minimum difference of 10% for the endpoint (IR) between the study group and the control group. By assuming a mean of two embryos transferred per patient, a total of 328 embryos (164 patients) were needed in each group.

### Statistical Methods

Chi-squared or Fisher’s exact tests were used for categorical variables where appropriate. An independent sample *t*-test was used for continuous variables that were normally distributed, and the Mann–Whitney *U*-test was used for data not normally distributed. Data are presented as means ± SD unless otherwise stated. Multivariate logistic regression analyses to determine the effect of the GnRHa on the OP and LB was performed in the GnRHa arm. Potential predictive factors were identified by univariate logistic regression per candidate predictive factor using a *P* value of <0.2 as a criterion for exclusion. Candidate predictive factors that were evaluated including age, basal LH, prolactin on day 2 of the cycle, duration of stimulation, total dose of r-FSH, total dose of antagonist, ovarian response to stimulation, E2 and LH levels on the day of triggering, embryo quality, number of transferred embryos, mid luteal LH level, and the number of vitrified embryos. The ovarian response was dichotomized into two groups, in order to simplify the interpretation and application of the model, according to the number of follicles ≥11 mm observed on the day of trigger (20): normal responders (NR; ≤13 follicles) and high responders (HR; 14–30 follicles). Out of all 328 patients, 178 were NR and 150 were HR. In the GnRHa arm, 100 patients were NR and 65 were HR. All the above covariates were examined for independence. The model was performed with the backward procedure. For both outcomes (OP and LB), the model is presented with the odds ratio (OR), 95% confidence interval (CI), and *P* value for each predictor. The area under the curve (AUC) was calculated to assess the model’s discriminative capacity. All *P*-values quoted are two-sided, and *P* < 0.05 was considered statistically significant. The SPSS statistical package (Release 24.0; SPSS, Inc.) was used for statistical evaluation.

## Results

**Figure 1:**
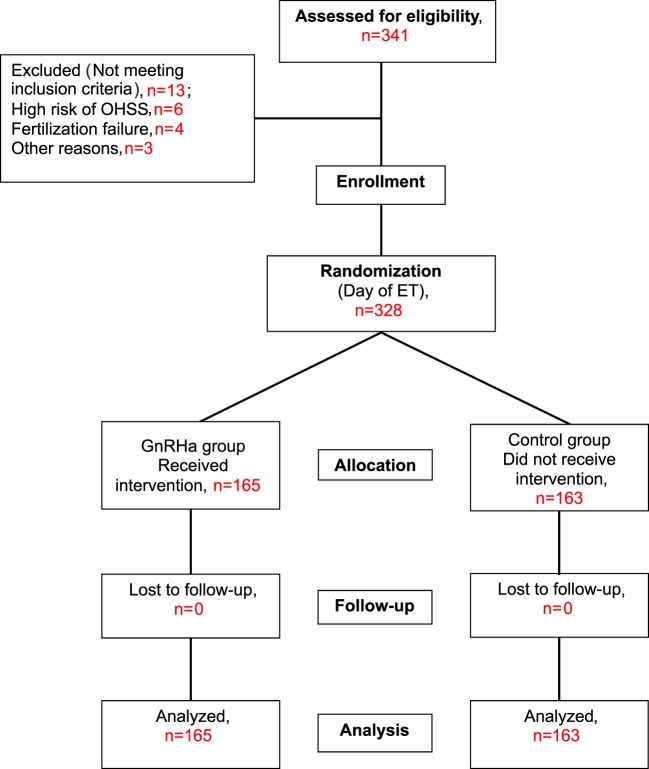
Study flow chart. Benmachiche. Mid-luteal GnRHa in GnRHa triggered cycles.

The trial was performed according to CONSORT guidelines. The flow of participants is shown in Figure 1. A total of 341 patients met the study criteria and were recruited. However, 13 patients were subsequently excluded, 6 patients due to hyper-responsiveness to stimulation (>30 follicles), 4 patients due to fertilization failure or failed embryo development, and 3 patients for other reasons. Therefore, a total of 328 patients were randomized into the study group (*n* = 165) or the control group (*n* = 163) on the day of ET. None of these patients were lost at follow-up; therefore, a total of 165 patients in the study group and 163 patients in the control group were included in the main analyses.

### Demographic Data and Cycle Characteristics

The baseline characteristics of the patients and the outcomes of ovarian stimulation are shown in (Table 1). No significant differences were seen between the study and the control group. Fertilization rates and the number and quality of embryos transferred were also similar in both groups (Table 1).

**Table 1:** Baseline characteristics and stimulation outcome.

Variable	GnRHa (OPU + 6)	Control	*P* value
Patients, *n*	165	163	
Age (years)	31.7 ± 3.6	32.1 ± 4.1	NS
Body mass index (kg/m^2^)	27.8 ± 4.8	28.2 ± 4.5	NS
Duration of follicle-stimulating hormone (FSH) stimulation (days)	9.1 ± 1.2	9.3 ± 1.1	NS
Total dose of FSH (IU)	1,831.1 ± 317.0	1,844.3 ± 294.7	NS
Total dose of antagonist (mg)	0.9 ± 0.2	1.0 ± 0.2	NS
Endometrium (mm)	10.7 ± 1.4	10.3 ± 1.5	NS
Follicles the day of triggering, *n*	12.2 ± 5.8	13.5 ± 6.4	NS
Oocytes, *n*	9.0 ± 4.5	9.9 ± 5.4	NS
MII oocytes, *n*	7.2 ± 3.8	8.1 ± 4.8	NS
2PN zygotes, *n*	5.4 ± 2.8	6.1 ± 3.7	NS
Embryos, *n*	5.1 ± 2.8	5.7 ± 3.4	NS
Transferred embryos, *n*	2.3 ± 0.5	2.4 ± 0.8	NS
Vitrified embryos, *n*	0.7 ± 1.6	0.9 ± 1.8	NS

*Values are means ± SD. NS = Not significant*.

*GnRHa, gonadotropin-releasing hormone agonist; MII, metaphase II; PN, pro-nuclei*.

### Serum Hormone Levels

Endocrine profiles are depicted in Table 2. Serum levels of FSH, LH, and E2 at the start (day 1 of stimulation) and at the end of ovarian stimulation were similar in treatment and control groups. However, serum concentrations of FSH, LH, P, and E2 during the LP on OPU + 7 were significantly higher in the GnRHa-supplemented group compared to the control group.

**Table 2:** Endocrine parameters.

Variable	GnRHa (OPU + 6)	Control	*P* value
Patients, *n*	165	163	
Basal FSH (IU/L)	5.9 ± 1.7	5.8 ± 1.7	0.32
Basal LH (IU/L)	5.1 ± 3.3	5.2 ± 3.1	0.67
Basal E2 (pg/mL)	42.0 ± 20.9	58.8 ± 198.0	0.28
Basal Prolactin (ng/mL)	21.5 ± 19.4	20.9 ± 12.7	0.74
LH (IU/L) day of triggering	1.3 ± 1.0	1.2 ± 0.8	0.40
P (ng/mL) day of triggering	1.1 ± 0.6	1.1 ± 0.6	0.92
E2 (pg/mL) day of triggering	1,852.7 ± 889.7	2,034.8 ± 917.6	0.07
FSH OPU + 7 (IU/L)^a^	0.9 ± 0.4	0.6 ± 0.3	0.001
LH OPU + 7 (IU/L)^a^	4.1 ± 2.1	1.4 ± 1.4	0.001
E2 OPU + 7 (pg/mL)^a^	1,274.3 ± 861.1	971.4 ± 849.6	0.001
P OPU + 7 (ng/mL)^a^	53.3 ± 33.5	37.6 ± 23.0	0.001
β-hCG OPU +14 (mIU/mL)	245.3 ± 377.0	191.7 ± 342.1	0.17

*Values are mean ± SD, unless otherwise noted*.

*^a^Statistically significant difference between groups, with P < 0.05*.

*OPU, oocyte pick-up; FSH, follicle-stimulating hormone; LH, luteinizing hormone; P, progesterone; E2, estradiol; hCG, human chorionic gonadotropin*.

### Reproductive Outcomes

No significant differences were observed in IRs [27 vs. 23% OR 1.2 (95% CI 0.9–1.7)], percentage of positive pregnancy tests [46 vs. 42% OR 1.2 (95% CI 0.7–1.8)], clinical pregnancy rate [38 vs. 31% OR 1.3 (95% CI 0.8–2.1)], OP rate [37 vs. 31% OR 1.3 (95% CI 0.8–2.1)], LB rate [36 vs. 31% OR 1.3 (95% CI 0.8–2.0)], and early pregnancy loss rate [16 vs. 27% OR 0.5 (95% CI 0.2–1.2)], in the study and control group, respectively (Table 3). The results of the Multivariate logistic regression analysis are given in (Table 4). In the luteal GnRHa arm, 62/165 (37.6%) patients had an OP and 60/165 (36.4%) had a LB. By studying, the coefficients related to each explanatory variable individually, at a risk of 5% we found that five factors increased the likelihood of OP: normo-responders (NR), higher pre-ovulatory LH levels, higher mid-luteal LH levels, good embryo quality, and a higher number of embryos transferred. The same factors, except for the number of transferred embryos, had a predictive value on the LB. The areas under the receiver operating characteristic (ROC) curve for OP and LB were 0.80 and 0.70, respectively.

**Table 3:** Overall reproductive outcome.

Variable	GnRHa (OPU + 6)	Control	OR (95% CI)	*P* value
Patients, *n*	165	163		
Positive pregnancy, *n* (%)	76/165 (46)	69/163 (42)	1.2 (0.7–1.8)	0.49
Implantation rate, *n* (%)	104/381 (27)	92/386 (23)	1.2 (0.9–1.7)	0.27
Clinical pregnancy rate, *n* (%)	63/165 (38)	51/163 (31)	1.3 (0.8–2.1)	0.19
Ongoing pregnancy rate, *n* (%)	62/165 (37)	51/163 (31)	1.3 (0.8–2.0)	0.23
Delivery rate, *n* (%)	60/165 (36)	50/163 (31)	1.3 (0.8–2.0)	0.27
Early miscarriage rate, *n* (% of positive hCG)	13/76 (17)	19/69 (27)	0.5 (0.2–1.2)	0.13
OHSS^a^, *n* (%)	1/165 (0.6)	1/163 (0.6)	1.0 (0.1–16.0)	0.99

*Values are number (percentage). There were no significant differences between the two groups*.

*^a^Moderate late ovarian hyper stimulation syndrome (OHSS)*.

**Table 4:** Multivariate regression results: the effect of mid luteal GnRHa on ongoing pregnancy (OP) and live birth (LB): patients in the GnRHa arm.

Variable	OR	(95% CI)	*P* Value	OR adj.	(95% CI)	*P* value
		Univariate analysis			Multivariate analysis	
**OP^a^**						
Basal LH (mIU/mL)	0.90	0.80–1.02	0.11	0.86	0.74–1.01	0.08
Total dose of r-FSH (IU)	1.00	1.000–1.002	0.07	1.001	1.000–1.002	0.06
Ovarian response status (NR vs. HR)	2.61	1.31–5.20	0.006	3.18	1.41–7.17	0.005
LH (mIU/mL) day of triggering	1.34	0.98–1.83	0.06	1.75	1.16–2.63	0.007
LH (mIU/mL) day OPU + 7	1.15	0.89–1.33	0.08	1.25	1.04–1.50	0.01
Embryo quality (Good vs. Bad)	2.40	0.91–6.30	0.08	3.67	1.22–11.06	0.02
Transferred embryos, *n*	1.63	0.92–2.91	0.09	2.11	1.07–4.19	0.03

**LB^b^**						
Ovarian response status (NR vs. HR)	2.41	1.21–4.80	0.01	3.37	1.56–7.27	0.002
LH (mIU/mL) day of triggering	1.34	0.98–1.83	0.06	1.65	1.12–2.43	0.01
LH (mIU/mL) day OPU + 7	1.13	0.97–1.32	0.11	1.65	1.12–2.43	0.01
Embryo quality (Good vs. Bad)	2.25	0.85–5.93	0.10	3.53	1.24–10.05	0.01

*^a^The receiver operating characteristic curve (AUC) for OP: 0.80*.

*^b^The receiver operating characteristic curve (AUC) for LB: 0.70*.

*OR, odds ratio; OR adj, adjusted odds ratio; CI, confidence interval 95%; NR, normo responders; HR, high responders*.

### Ovarian Hyper Stimulation Syndrome

No early onset OHSS cases were seen in the two groups. No late onset OHSS cases were seen in NR patients (≤13 follicles). However, two moderate-late-onset OHSS cases occurred in the HR subgroup (14–30 follicles). One case occurred in the luteal GnRHa group, with the other in the control group. Both patients were shortly hospitalized (24–48 h) with only symptomatic relief.

## Discussion

This randomized controlled study explored the possible effect of a mid-luteal GnRHa bolus on IRs in IVF/ICSI patients. Overall, no significant differences were seen in implantation and clinical pregnancy rates. In contrast, significantly higher mid-luteal steroid levels were seen in the group of patients who received a mid-luteal bolus of GnRHa (Table 2). Two cases of moderate-late onset OHSS occurred; one case in the study group and one in the control group. Both of these cases were associated with twin pregnancies (Table 3).

Several studies have previously shown a positive effect of GnRHa administration during the LP. The evidence for its efficacy is, however, of low quality due to the heterogeneity between trials (21–24). In the present study, the IR, OP, and LB rates were slightly higher in the GnRHa group compared to the control group [27 vs. 23% OR 1.2 (95% CI 0.9–1.7), *P* < 0.27], [37 vs. 31% OR 1.3 (95% CI 0.8–2.1), *P* < 0.23], and [36 vs. 31% OR 1.3 (95% CI 0.8–2.0), *P* < 0.27], respectively; however, the differences fell short of reaching statistical significance (Table 3). However, an absolute risk difference of 5% in LB rate - although not significant - is clinically relevant. In fact, if sample size had been calculated taking OP as the main outcome, which could be regarded more relevant in IVF cycles than IR, we would have needed twice the number of patients included in this study and results might have been statistically significant in favor of the intervention group (at least this is the trend).

In 2004, Tesarik et al. demonstrated that a single supplementary bolus of a GnRHa as LPS in oocyte recipient cycles without pituitary desensitization-improved the IR (36.9 vs. 25.1%) and LB (31.1 vs. 21.5%). In 2006, the same investigators evaluated the effects of 0.1 mg triptorelin administration 6 days after ICSI in GnRHa (*n* = 300) and GnRH antagonist (*n* = 300) cycles. IRs were significantly increased with both regimens. However, only in GnRH antagonist cycles a significant increase in the OP was observed (16). Subsequent studies demonstrated that LP GnRHa supplementation seemed to benefit the GnRH antagonist co-treated cycle (8, 16, 25–27) more than the long GnRHa co-treated cycle (18, 19, 28). The main reason for this is the down-regulation of the pituitary, which is induced by the long GnRHa protocol (28). In support of this conclusion, Kung et al. (29) recently verified that luteal GnRHa administration significantly increased clinical pregnancy and LB rates in GnRH antagonist co-treated patients, but not in patients undergoing a long GnRHa down-regulation protocol. Interestingly, Kung et al. (29) suggested that patients with higher basal FSH levels (>8 mIU/mL) and reduced numbers of mature oocytes (≤3) might have better outcomes when receiving LP support with GnRHa.

In the present study, although we demonstrated a significant increase in mid-luteal endogenous gonadotropins and steroids in the study group, we failed to reject the null hypothesis in terms of implantation and pregnancy rates. A subsequent question remaining to be answered is if there were any confounding factors impacting the clinical effects of the intervention. To explore this issue, we conducted a multivariate regression analysis, exploring variables that could independently correlate with the occurrence of OP and LB in the luteal GnRHa arm. Implantation was not chosen as an outcome parameter of the model, as the transfer of more than one embryo could partially compensate for early pregnancy losses, moreover, the prediction of OP and LB was more clinically relevant. In the study population (GnRHa group), 37.6% (62/165) of women had an OP and 36.4% (60/165) had a LB. The results of the multivariate regression (Table 4) show that the ovarian response might confound the relationship between the mid-luteal GnRHa dose and the pregnancy outcome. The NR patient was three times more likely to have OP [*P* < 0.005, OR 3.18 (95% CI 1.41–7.17)] and LB [*P* < 0.002, OR 3.37 (95% CI 1.56–7.27)], respectively, compared to the HR patient. Indeed, the OP rate increased from 9.70% (16/165) in HR to 27.88% (46/165) in NR and the LB rate increased from 9.70% (16/165) in HR to 26.66% (44/165) in NR. The reason for this discrepancy may be attributed to a possible threshold of mid-luteal steroids compatible with optimal endometrial receptivity (30–35). Moreover, the availability of good embryo quality, a pre-ovulatory, and mid-luteal LH activities were also associated with increased odds for both OP and LB. Finally, as expected, the number of embryos transferred was independently associated with the occurrence of OP (Table 4). The areas under the ROC curve for OP and LB were 0.80 and 0.70, respectively, indicating that both models fit moderately well and are clinically useful, and as results provide a tool with which to inform patients of the prognosis after the mid luteal addition of the GnRHa. Thus, the addition of a mid-luteal bolus GnRHa to NR (<14 follicles) but not to HR (14–30) seems to be the fine-tuning sought to optimize the protocol suggested by Humaidan et al. (3, 4) in terms of reproductive outcome in both categories. Whereas, in the very HR (>30 follicles) category, which was excluded from the current study, the freeze all strategy remains best option in our opinion.

The exact mechanism behind the presumed beneficial effect of LP GnRHa administration remains poorly defined. It has been hypothesized that GnRHa either supports CL function by inducing LH secretion by the pituitary gonadotrophic cells (8, 26) or stimulates the endometrial GnRH receptors (36). Tesarik et al. (15) postulated a direct effect of GnRHa on the embryo, as suggested by an increase in β-HCG secretion. The latter mechanism does not seem to be supported by our study because there were no significant differences in hCG levels between study and control groups (Table 2). Interestingly, the findings of the present study show that a bolus of GnRHa, when administered 6 days after OPU in GnRH antagonist cycles, is able to induce a surge of pituitary gonadotropins (FSH and LH), eliciting an increase in steroid production (E2 and P) by the CL (Table 2). The above-mentioned hormone variations were still detectable within 24 h following the GnRHa administration; 14–16 h in our protocol precisely. Thus, the increase of FSH, LH, P, and E2 was significantly higher in the study group compared to the control group (Table 2), which is in agreement with previous reports (8, 26). However, similar changes in the hormonal luteal profile have not been reported in other studies (16, 37).

In terms of safety, we did not observe any increase in the development of OHSS in GnRHa-treated patients, which is in agreement with Tesarik et al. (16). Thus, this regimen could be considered a safe approach to support the LP with LH activity when comparing with mid-luteal addition of low dose of hCG (38).

The limitations of the present study are, firstly, from a clinical viewpoint, OP and LB are more relevant outcomes in IVF cycles than IR (39, 40). IR will equate with clinical pregnancy rate only when single embryo transfer (SET) is used (41). In the present study, only 5% (19/328) of cycles were SET cycles. If a clinical pregnancy was considered as the main outcome, the number of patients required would be twice that of the present study (more than 600), which would be beyond the capacity of a single fertility center. Secondly, as the secondary data analyzed used small numbers reported from a single center, this limited generalizability and so these data should be interpreted with caution and be considered only as preliminary to a larger future study.

In conclusion, the present study demonstrates that a single dose of GnRHa administered 6 days after OPU in GnRHa-triggered IVF cycles supplemented with a small bolus of hCG on the day of OPU did not improve reproductive outcomes in the study group as compared to controls, despite the fact that significantly higher mid-luteal endogenous gonadotropins and steroid levels were present in the study group. Results of the multivariate regression analysis performed in the luteal GnRHa population support the concept of “individualized” ovarian response-based luteal support, suggesting that the above-mentioned regimen appears to be a promising alternative to provide an optimal level of LH activity throughout the early-mid-LP to maximize each patient’s chance of a pregnancy with minimal safety issues. The findings need further corroboration in a large-scale study.

## Ethics Statement

All patients provided written informed consent to participate in the study, which was conducted in accordance with the Declaration of Helsinki and Good Clinical Practice. The research project was approved by the Ethics Committee of the University hospital Centre Ibn Badis, Constantine. Moreover, the study was registered in ClinTrial.gov, Number: 02053779.

## Author Contributions

ABenmachiche and PH designed the study, drafted, and edited the manuscript. ABenmachiche performed data collection, handling of data, and statistical analysis. AZ, SB, and ABoularak actively participated in data acquisition, handling of data and manuscript drafting. All co-authors accepted the final draft.

## Conflict of Interest Statement

ABenmachiche, SB, AZ, and ABoularak have nothing to disclose. PH reports personal fees from Merck, MSD, and IBSA, grants from Merck, MSD, and Ferring, outside the submitted work.

## Abbreviations

GnRHa, GnRH agonist; IVF, *in vitro* fertilization; ICSI, intracytoplasmic sperm injection; FSH, follicle-stimulating hormone; LH, luteinizing hormone; HCG, human chorionic gonadotropin; LPS, luteal phase support; IR, implantation rate; OP, ongoing pregnancy; LB, live birth; OHSS, ovarian hyperstimulation syndrome; E2, estradiol; P, progesterone; CIs, confidence intervals; AUC, the area under the receiver operating characteristic (ROC) curve.
